# The joint influence of area income, income inequality, and immigrant density on adverse birth outcomes: a population-based study

**DOI:** 10.1186/1471-2458-9-237

**Published:** 2009-07-14

**Authors:** Nathalie Auger, Julie Giraud, Mark Daniel

**Affiliations:** 1 Unité Études et analyses de l'état de santé de la population, Institut national de santé publique du Québec, Montréal, Québec, Canada; 2 Centre de recherche du Centre hospitalier de l'Université de Montréal, Montréal, Québec, Canada; 3 Département de médecine sociale et préventive, Université de Montréal, Montréal, Québec, Canada; 4 Institut de Santé Publique d'Épidémiologie et de Développement, Bordeaux School of Public Health, Bordeaux, France; 5 School of Health Sciences, University of South Australia, Adelaide, Australia

## Abstract

**Background:**

The association between area characteristics and birth outcomes is modified by race. Whether such associations vary according to social class indicators beyond race has not been assessed.

**Methods:**

This study evaluated effect modification by maternal birthplace and education of the relationship between neighbourhood characteristics and birth outcomes of newborns from 1999–2003 in the province of Québec, Canada (N = 353,120 births). Areas (N = 143) were defined as administrative local health service delivery districts. Multi-level logistic regression was used to model the association between three area characteristics (median household income, immigrant density and income inequality) and the two outcomes preterm birth (PTB) and small-for-gestational age (SGA) birth. Effect modification by social class indicators was evaluated in analyses stratified according to maternal birthplace and education.

**Results:**

Relative to the lowest tertile, high median household income was associated with SGA birth among Canadian-born mothers (odds ratio (OR) 1.13, 95% confidence interval (CI) 1.06, 1.20) and mothers with high school education or less (OR 1.13, 95% CI 1.02, 1.24). Associations between median household income and PTB were weaker. Relative to the highest tertile, low immigrant density was associated with a lower odds of PTB among foreign-born mothers (OR 0.79, 95% CI 0.63, 1.00) but a higher odds of PTB among Canadian-born mothers (OR 1.14, 95% CI 1.07, 1.21). Associations with income inequality were weak or absent.

**Conclusion:**

The association between area factors and birth outcomes is modified by maternal birthplace and education. Studies have found that race interacts in a similar manner. Public health policies focussed on perinatal health must consider the interaction between individual and area characteristics.

## Background

A growing number of studies have reported associations between area-based characteristics and perinatal health. Research has focussed on neighbourhood economic deprivation in relation to gestational age [1-8] and birthweight [5,7,9-14]. Neighbourhood deprivation has been found to have an unfavourable association with these birth outcomes, even accounting for individual socio-economic status. Some studies have focussed on neighbourhood segregation and gestational age [15-19] or birthweight [15,16,19-21], but the evidence is conflicting. Studies demonstrate both protective [16,19] and unfavourable [15-18,20-22] associations with birth outcomes.

Recent work has evaluated associations between area characteristics and birth outcomes across different social classes, with particular attention on ethnic segregation and racial groups in the US [8,15,17-21]. The evidence suggests that African-American and white mothers may be differentially susceptible to segregation, although study results are not consistent [17,20]. A small number of studies have found that area deprivation also interacts with race [5,6,8,20].

While such research attests to a contextual influence that varies among US racial classes, information for other nations and social markers is lacking. In other settings, measures other than race alone may better represent social differences. Whether the influence of area characteristics varies according to such factors has not been evaluated. Some evidence suggests that the influence of area-based economic deprivation may vary depending on maternal foreign-birth status [7].

These knowledge gaps also apply to area characteristics emerging as predictors of birth outcomes, such as income inequality. Measures of income inequality represent the unequal distribution of income within areas [23], in contrast to area poverty measures which reflect mean income [24]. Although controversial, income inequality is recognised as a determinant of mortality [25], and, possibly, of birth outcomes [23,26]. Studies have focussed on state or country-level income inequality, rather than small areas where evidence has been less compelling [27]. However, an adverse influence of income inequality on preterm birth (PTB) was reported in a recent study of US counties [23]. The investigators found that the influence of income inequality depended on race [23]. A separate study on cumulative exposure to income inequality reported an association with PTB for Hispanic but not Black or White ethnicity [28]. No study outside the US has yet examined birth outcomes and income inequality.

The study of place-based influences is also challenged by the complex relationships between contextual factors and health outcomes [29]. Income inequality might be a pathway through which area income exerts its effects [24], or might independently influence birth outcomes. One way to consider these issues is to examine the joint influence of different area characteristics [26]. Area deprivation and segregation have been investigated together in a limited number of studies [5,17,20]. No study has considered income inequality together with other area variables in relation to birth outcomes.

These gaps in the literature are important to address as public health interventions often focus on sub-groups at risk, an approach that may be justified by the limited resources available for interventions. An important first challenge is to identify the appropriate sub-groups, and then act to reduce the inequalities present within these groups.

The objective of this study was to examine the association between birth outcomes and area income, income inequality and segregation across different social markers in Québec, Canada. Québec is a province with a supportive social welfare system and universal health care [7], and can be considered more egalitarian than US states [30]. The outcomes analysed were PTB and small-for-gestational-age (SGA) birth. PTB is responsible for a large proportion of infant morbidity and mortality [6]. SGA birth is useful as measure of fetal growth restriction [31,32]. We did not consider low birth weight [32]. We evaluated whether the relationship between area characteristics and these birth outcomes was modified by two social markers, maternal birthplace and education.

## Methods

### Data

Data were obtained from the 1) Québec birth file and 2) 2001 Canada census. Singleton births for a 5-year period (1999–2003) centered on the 2001 Census year were extracted (N = 356,147). Residential area at time of birth was specified as administrative local community service center (CLSC) districts (N = 166). CLSCs are areas of health service delivery [33] where variability in area-deprivation has been previously documented [34]. CLSCs are comparable to neighbourhoods as their borders are based on historically defined neighbourhoods, integrate geographic or natural boundaries, and their residents report a subjective sense of place [35]. We examined a fused version of CLSCs provided by the health ministry (N = 147) to ensure areas were sufficiently large for the calculation of income inequality [mean population 51,564 (range 3,700 to 142,025); mean births 2,469 (range 230 to 8,674)]. Four northern CLSCs (representing 3,027 births) were excluded because reliable census data were not available for these areas. The final dataset was hierarchically structured with 353,120 births nested in 143 CLSCs.

### Variables

Outcomes were dichotomously coded and defined as 1) PTB, less than 37 weeks gestation, and 2) SGA birth (birthweight less than 10^th ^percentile for age and sex for infants from 22 to 43 weeks gestation) [31]. Extreme cases of PTB (N = 50, defined as less than 20 weeks gestation) were excluded as these likely represent implausible values [36]. Gestational age was missing for 64 births; SGA status could not be calculated for 393 births because gestational age was either missing or not within 22 to 43 weeks. One final file for each outcome was created after exclusion of extreme and missing data on PTB and SGA birth (N_PTB_= 353,006; N_SGA _= 352,727).

Individual-level covariates included maternal education (no high school diploma, high school diploma, some post-secondary, some university or more, or unknown, representing less than 11, 11, 12–13, and 14+ years of education, respectively), birthplace (foreign-born, Canadian-born, or unknown), age (less than 20, 20–34, or 35+ years), civil status (married, cohabiting, single, or unknown), previous live births (none, one or more), and sex of infant. These factors have been previously shown to be associated with adverse birth outcomes [4].

Three area-level variables were calculated, using aggregated 2001 Canada Census data for household income and immigration status: 1) Median household income, used as an indicator of neighbourhood poverty/economic deprivation; 2) Proportion immigrant population, used as an indicator of immigrant density; 3) Coefficient of variation (CV) of mean household income, used as an indicator of income inequality. The CV was calculated according to standard methods, after verification that household income was normally distributed [37]. Large CVs indicate areas with greater inequality. Tertiles of neighbourhood variables were examined. Each tertile contained approximately one-third of births.

### Statistical analysis

Descriptive analyses were performed, and trends were examined using the Cochran-Armitage test. Because the data were hierarchically structured, we used multi-level multivariate logistic regression with CLSC specified as a random effect to model the association between area variables and birth outcomes [38]. Multi-level analyses consisted of a set of three models, one for the crude association between area variables and birth outcomes, a second adjusting for individual-level covariates (maternal education, birth place, age, civil status, previous births, infant sex), and a third adjusting for individual and all three neighbourhood variables. We estimated odds ratios (OR) and 95% confidence intervals (CI). Statistical significance was determined with the Wald statistic. Models for SGA birth did not adjust for sex of infant because sex is accounted for in the calculation of SGA percentile [31]. Missing data for independent variables were included as separate categories in analyses.

To examine the influence of area variables on birth outcomes across social markers potentially serving as effect modifiers, models were re-run after stratifying data according to maternal birthplace (foreign- *versus *Canadian-born) and education (high school diploma or less *versus *some post-secondary or more).

Final models were run a second time using Generalised Estimating Equations (GEE) for correlated observations with a logit link [39]. Both multi-level models and GEE have been used in the literature to evaluate neighbourhood effects [23,40]. Multi-level models yielded similar ORs and CIs as GEE models, and low intraclass correlations (PTB 0.0048, SGA birth 0.0032 – calculated using the latent variable method [41]). Where convergence of multi-level models did not occur due to limited between-cluster variation, we provide results for GEE models. GEE invokes a population-averaged estimate (or marginal mean) not matched on cluster, and overcomes convergence problems by estimating the within cluster similarity of residuals as a basis for regression parameters and standard errors [42]. In contrast, multi-level models parameterize the mean response conditional on random effects and model the between cluster variation [42].

SAS 9.1 software was used for analyses (SAS Institute Inc, Cary, NC, 2002). Multi-level models were run using the GLIMMIX macro [43], and GEE models using the REPEATED statement of PROC GENMOD. This study was conducted as part of the Québec population health surveillance plan mandated by the health ministry and approved by the Public Health Ethics Committee.

## Results

### Descriptive characteristics

Areas had a mean CV of 18.5% (range 2.3%–47.1%), mean median household income of $41,352 (range $23,891– $79,163), and mean immigrant proportion of 7.7% (range 0%–62.0%). PTB and SGA birth represented 6.3% and 8.3% of births, respectively (Table 1). PTB and SGA birth were progressively prevalent among mothers with lesser levels of education. The frequency of PTB among foreign-born mothers was similar to native-born mothers (6.3%), but SGA birth was slightly more common in foreign-born (9.5%) than native-born (8.1%) mothers. PTB and SGA birth were positively related to area poverty and inversely related to income inequality. While PTB was slightly more frequent in areas with low immigrant density, SGA birth was more frequent in areas with high immigrant density (Table 1).

**Table 1:** Frequency of preterm and small-for-gestational age birth according to individual- and area-level characteristics, singleton births, Québec, Canada, 1999–2003

	**PTB**		**SGA birth**	
	**%**	N*	**%**	N*
		
**Individual-level characteristics**				
**Maternal birth place**				
Foreign-born	6.3	59 474	9.5	59 432
Canadian-born	6.3	291 149	8.1	290 922
**Maternal education**				
No high school diploma	8.3	41 558	12.0	41 528
High school diploma	7.3	35 473	10.0	35 450
Some post secondary	6.3	67 391	8.9	67 343
Some university or more	5.4	183 041	6.8	182 918
**Maternal age**				
<20 years	8.8	14 236	11.7	14 222
20–34 years	6.1	289 745	8.1	289 529
35+ years	7.0	49 025	8.6	48 976
**Civil status**				
Married	5.7	146 691	7.3	146 590
Cohabiting	6.4	172 401	8.6	172 268
Single	8.9	24 870	11.8	24 840
**Previous births**				
None	7.2	167 749	10.4	167 613
One or more	5.5	185 257	6.5	185 114
**Infant sex**				
Male	6.7	181 422	8.4	181 279
Female	5.9	171 584	8.3	171 448
**Area-level characteristics**				
**Income**				
Poorest	6.7	114 187	9.0	114 076
Intermediate	6.4	119 311	8.4	119 223
Wealthiest	5.9	119 508	7.6	119 428
**Immigrant density**				
Low	6.6	115 375	8.1	115 287
Intermediate	6.2	119 046	8.0	118 956
High	6.2	118 585	8.9	118 484
**Income inequality**				
High	6.1	117 485	8.1	117 386
Intermediate	6.3	118 209	8.3	118 120
Low	6.6	117 312	8.5	117 221
**Total**	6.3	353 006	8.3^†^	352 727

PTB = preterm birth; SGA = small-for-gestational-age

* Denominators do not sum to total because missing data are not shown.

† The overall prevalence of SGA birth has declined in Québec in the past decade, which explains the 8.3% prevalence rather than the expected 10% (the SGA reference curves were constructed for 1994–1996 at a time when SGA birth was more common)[31].

Higher area poverty was associated with progressively higher proportions PTB and SGA birth for most social markers (*i.e*., foreign-born, native-born, lesser education, higher education, Table 2). A statistically non-significant exception was the trend for PTB among foreign-born mothers.

**Table 2:** Distribution of adverse birth outcomes according to area characteristics, maternal birth place and education, singleton births, Québec, Canada, 1999–2003

	**Foreign-born**		**Canadian-born**		**High school diploma or less**		**Some postsecondary or more**	
**PTB**	% PTB	N	% PTB	N	% PTB	N	% PTB	N
		
**Area income**								
Poorest	6.4	21 657	6.7	91 628	8.1	31 393	5.9	75 344
Intermediate	6.3	22 312	6.4	96 177	7.9	25 718	5.7	84 234
Wealthiest	6.0	15 505	5.9	103 344	7.4	19 920	5.4	90 854
Trend *p*-value	0.07		<.0001		0.004		<.0001	
**Immigrant density**								
Low	4.9	2 043	6.6	112 714	8.1	27 536	6.0	82 546
Intermediate	5.6	7 579	6.2	111 006	7.7	22 350	5.5	86 045
High	6.4	49 852	6.0	67 429	7.6	27 145	5.4	81 841
Trend *p*-value	0.0002		<.0001		0.01		<.0001	
**Income inequality**								
High	6.3	35 819	6.0	80 636	7.3	22 161	5.5	86 780
Intermediate	6.0	16 783	6.3	100 812	8.0	25 257	5.6	84 382
Low	6.3	6 872	6.6	109 701	8.0	29 613	5.9	79 270
Trend *p*-value	0.28		<.0001		0.004		0.002	
**SGA birth**	% SGA	N	% SGA	N	% SGA	N	% SGA	N
		
**Area income**								
Poorest	10.0	21 639	8.8	91 542	11.5	31 365	7.9	75 291
Intermediate	9.4	22 301	8.2	96 102	11.0	25 703	7.5	84 175
Wealthiest	9.1	15 492	7.3	103 278	10.5	19 910	6.8	90 795
Trend *p*-value	0.0009		<.0001		0.0002		<.0001	
**Immigrant density**								
Low	9.0	2 042	8.1	112 628	10.8	27 514	7.2	82 492
Intermediate	8.7	7 573	7.9	110 924	11.3	22 335	7.0	85 983
High	9.7	49 817	8.2	67 370	11.1	27 129	8.0	81 786
Trend *p*-value	0.006		0.33		0.20		<.0001	
**Income inequality**								
High	9.4	35 801	7.6	80 560	10.7	22 149	7.3	86 716
Intermediate	9.3	16 763	8.1	100 746	11.1	25 241	7.4	84 326
Low	10.7	6 868	8.4	109 616	11.3	29 588	7.4	79 219
Trend *p*-value	0.008		<.0001		0.03		0.22	

PTB = preterm birth; SGA = small-for-gestational-age

Greater immigrant density was negatively associated with PTB except among foreign-born mothers, for whom the relationship was positive (Table 2). For SGA birth, a pattern was observed among foreign-born mothers and mothers with higher education (greater immigrant density was associated with progressively higher proportions SGA birth).

Income inequality was inversely associated with PTB and SGA birth for most social markers, with the exception of PTB among foreign-born mothers and SGA birth among mothers with higher education (Table 2). A less pronounced trend (*P*-value = 0.03) for income inequality and SGA birth was present among mothers with lesser education.

### Non-stratified regression models

All area variables were associated with PTB in unadjusted models (Table 3). Relative to opposite tertiles, higher odds of PTB were found for high area poverty (OR 1.13, 95% CI 1.07–1.20) and low immigrant density (OR 1.07, 95% CI 1.01–1.14). Lower odds of PTB were found for high income inequality (OR 0.92, 95% CI 0.86–0.98). Associations remained after adjustment for individual-level variables, with the exception of income inequality (OR 0.96, 95% CI 0.90–1.01). In fully adjusted models (accounting for all three area variables), only immigrant density remained statistically significant (OR 1.10, 95% CI 1.02–1.18).

**Table 3:** Crude and adjusted associations between area characteristics and adverse birth outcomes, singleton births, Québec, Canada, 1999–2003

	**Crude OR**	**95% CI**	Adjusted OR*	**95% CI**	**Adjusted OR^†^**	**95% CI**
			
**PTB**						
**Area income**						
Poorest	1.13^‡^	1.07–1.20	1.06^‡^	1.01–1.12	1.05	0.98–1.12
Intermediate	1.08^‡^	1.02–1.14	1.04^‡^	0.99–1.10	1.02	0.96–1.09
Wealthiest	Referent		Referent		Referent	
**Immigrant density**						
Low	1.07^‡^	1.01–1.14	1.11^‡^	1.05–1.17	1.10	1.02–1.18
Intermediate	0.99^‡^	0.94–1.06	1.03^‡^	0.98–1.08	1.04	0.97–1.12
High	Referent		Referent		Referent	
**Income inequality**						
High	0.92	0.86–0.98	0.96^‡^	0.90–1.01	1.01	0.94–1.09
Intermediate	0.94	0.89–1.00	0.96^‡^	0.91–1.01	0.99	0.93–1.05
Low	Referent		Referent		Referent	
**SGA birth**						
**Area income**						
Poorest	1.21^‡^	1.14–1.29	1.09^‡^	1.03–1.14	1.09^‡^	1.03–1.15
Intermediate	1.12^‡^	1.05–1.19	1.05^‡^	1.00–1.10	1.06^‡^	1.00–1.12
Wealthiest	Referent		Referent		Referent	
**Immigrant density**						
Low	0.91^‡^	0.85–0.98	0.97	0.92–1.03	0.94^‡^	0.88–1.00
Intermediate	0.89^‡^	0.83–0.95	0.98	(0.92–1.05)	0.99^‡^	0.94–1.05
High	Referent		Referent		Referent	
**Income inequality**						
High	0.95	0.89–1.02	0.96	0.90–1.01	0.94^‡^	0.88–1.00
Intermediate	1.00	0.93–1.07	0.98	0.93–1.03	0.96^‡^	0.90–1.01
Low	Referent		Referent		Referent	

PTB = preterm birth; SGA = small-for-gestational-age

* Adjusted for individual-level variables (maternal age, education, civil status, birth place, and previous births). PTB is also adjusted for infant sex.

† Adjusted for individual-level variables and all variables in table.

‡ Results are for generalised estimating equations. All other results are for multi-level logistic regression models.

Unadjusted models indicated greater odds of SGA birth for high area poverty (OR 1.21, 95% CI 1.14–1.29), lower odds for low immigrant density (OR 0.91, 95% CI 0.85–0.98), and no association for high income inequality (OR 0.95, 95% CI 0.89–1.02), relative to the opposite tertile (Table 3). Upon adjustment for individual and area variables, the strength of associations with SGA birth diminished for area poverty (OR 1.09, 95% CI 1.03–1.15) and immigrant density (OR 0.94, 95% CI 0.88–1.00), but remained stable for income inequality (OR 0.94, 95% CI 0.88–1.00).

### Regression models stratified by maternal birth place

Among native born-mothers, high area poverty (OR 1.07, 95% CI 1.00–1.14) and low immigrant density (OR 1.14, 95% CI 1.07–1.21), but not income inequality (OR 1.02, 95% CI 0.95–1.09), were associated with PTB in the fully adjusted model (Table 4). Income inequality was nonetheless associated with PTB among native-born mothers in the model adjusted for individual-level variables only (OR 0.92, 95% CI 0.86–0.98). Among foreign-born mothers, only immigrant density was associated with PTB in the fully adjusted model (OR 0.79, 95% CI 0.63–1.00), with odds in the opposite direction than those of native-born mothers.

**Table 4:** Adjusted associations between area characteristics and adverse birth outcomes according to maternal birth place, singleton births, Québec, Canada, 1999–2003

	**Foreign-born**				**Canadian-born**			
	OR*	**95% CI**	**OR^†^**	**95% CI**	OR*	**95% CI**	**OR^†^**	**95% CI**
				
**PTB**								
**Area income**								
Poorest	0.96	0.85–1.09	0.95	0.83–1.09	1.07	1.02–1.13	1.07^‡^	1.00–1.14
Intermediate	0.96	0.85–1.09	0.95	0.83–1.08	1.04	0.98–1.11	1.03^‡^	0.97–1.09
Wealthiest	Referent		Referent		Referent		Referent	
**Immigrant density**								
Low	0.80	0.65–0.99	0.79	0.63–1.00	1.12	1.06–1.19	1.14^‡^	1.07–1.21
Intermediate	0.91	0.81–1.03	0.89	0.77–1.02	1.05	0.99–1.12	1.08^‡^	1.02–1.15
High	Referent		Referent		Referent		Referent	
**Income inequality**								
High	1.05	0.92–1.21	0.98	0.84–1.15	0.92	0.86–0.98	1.02^‡^	0.95–1.09
Intermediate	0.97	0.84–1.12	0.93	0.79–1.08	0.96	0.90–1.01	1.01^‡^	0.96–1.07
Low	Referent		Referent		Referent		Referent	
**SGA birth**								
**Area income**								
Poorest	1.02	0.92–1.14	1.00	0.88–1.13	1.11	1.05–1.17	1.13	1.06–1.20
Intermediate	0.95	0.85–1.06	0.93	0.83–1.05	1.06	1.00–1.13	1.08	1.02–1.15
Wealthiest	Referent		Referent		Referent		Referent	
**Immigrant density**								
Low	0.98	0.83–1.16	0.96	0.80–1.16	0.98	0.92–1.04	0.94	0.88–1.01
Intermediate	0.95	0.85–1.06	0.93	0.83–1.05	0.99	0.93–1.06	1.01	0.95–1.08
High	Referent		Referent		Referent		Referent	
**Income inequality**								
High	0.95	0.84–1.07	0.94	0.82–1.09	0.95	0.90–1.01	0.95	0.88–1.02
Intermediate	0.94	0.82–1.06	0.93	0.81–1.06	0.99	0.94–1.05	0.98	0.92–1.04
Low	Referent		Referent		Referent		Referent	

PTB = preterm birth; SGA = small-for-gestational-age

* Adjusted for individual-level variables (maternal age, education, civil status, birth place, and previous births). PTB is also adjusted for infant sex.

† Adjusted for individual-level variables and all variables in table.

‡ Results are for generalised estimating equations. All other results are for multi-level logistic regression models.

Among native-born mothers, SGA birth was more strongly associated with area poverty (OR 1.13, 95% CI 1.06–1.20) than immigrant density (OR 0.94, 95% CI 0.88–1.01) or income inequality (OR 0.95, 95% CI 0.88–1.02) in the fully adjusted model (Table 4). Among foreign-born mothers, area variables were not associated with SGA birth.

### Regression models stratified by maternal education

Among mothers with some postsecondary education or more, areas with low relative to high immigrant density were associated with unfavourable odds of PTB (OR 1.15, 95% CI 1.06–1.25) in the fully adjusted model (Table 5). An association was not present among mothers with a high school diploma or less (OR 1.01, 95% CI 0.90–1.12). Area poverty and income inequality were not associated with PTB for either social marker in the fully adjusted model.

**Table 5:** Adjusted associations between area characteristics and adverse birth outcomes according to maternal education, singleton births, Québec, Canada, 1999–2003*

	**High school diploma or less**				**Some postsecondary or more**			
	**OR^†^**	**95% CI**	**OR^‡^**	**95% CI**	**OR^†^**	**95% CI**	**OR^‡^**	**95% CI**
				
**PTB**								
**Area income**								
Poorest	1.08	0.99–1.18	1.08	0.97–1.19	1.04	0.97–1.12	1.02	0.95–1.10
Intermediate	1.07	0.98–1.18	1.08	0.97–1.19	1.02	0.95–1.09	0.99	0.92–1.07
Wealthiest	Referent		Referent		Referent		Referent	
**Immigrant density**								
Low	1.02	0.93–1.12	1.01	0.90–1.12	1.16	1.08–1.24	1.15	1.06–1.25
Intermediate	0.99	0.90–1.09	1.01	0.90–1.12	1.07	1.10–1.15	1.08	1.00–1.17
High	Referent		Referent				Referent	
**Income inequality**								
High	0.94	0.86–1.03	0.96	0.86–1.08	0.92	0.86–0.99	1.00	0.92–1.09
Intermediate	1.01	0.94–1.10	1.03	0.94–1.13	0.92	0.86–0.98	0.97	0.90–1.04
Low	Referent		Referent		Referent		Referent	
**SGA birth**								
**Area income**								
Poorest	1.09	1.00–1.19	1.13	1.02–1.24	1.08	1.03–1.14	1.07	1.00–1.13
Intermediate	1.05	0.96–1.14	1.09	0.99–1.20	1.05	1.00–1.11	1.05	0.99–1.11
Wealthiest	Referent		Referent		Referent		Referent	
**Immigrant density**								
Low	0.95	0.87–1.04	0.90	0.81–0.99	1.00	0.94–1.05	0.95	0.89–1.02
Intermediate	1.02	0.93–1.12	1.04	0.94–1.15	0.97	0.91–1.03	0.97	0.91–1.04
High	Referent		Referent		Referent		Referent	
**Income inequality**								
High	0.96	0.88–1.05	0.92	0.83–1.03	0.94	0.89–0.99	0.93	0.87–1.00
Intermediate	0.98	0.90–1.06	0.94	0.86–1.03	0.98	0.93–1.03	0.97	0.92–1.03
Low	Referent		Referent		Referent		Referent	

PTB = preterm birth; SGA = small-for-gestational-age

* All results are for multi-level logistic regression models.

† Adjusted for individual-level variables (maternal age, education, civil status, birth place, and previous births). PTB is also adjusted for infant sex.

‡ Adjusted for individual-level variables and all variables in table

A different pattern was present for SGA birth. Among mothers with some postsecondary education or more, high relative to low area poverty was associated with unfavourable odds of SGA birth (OR 1.07, 95% CI 1.00–1.13), and high relative to low income inequality was associated with lower odds of SGA birth (OR 0.93, 95% CI 0.87–1.00) in the fully adjusted model (Table 5). Among mothers with a high school diploma or less, area poverty was more strongly associated with SGA birth (OR 1.13, 95% CI 1.02–1.24); high income inequality was still protective, albeit not statistically significant (OR 0.92, 95% CI 0.83–1.03). Unlike PTB, immigrant density was not associated with SGA birth among mothers with some postsecondary education or more. However, among mothers with a high school diploma or less, low relative to high immigrant density was protective for SGA birth (OR 0.90, 95% CI 0.81–0.99) in the fully adjusted model.

## Discussion

This study examines, in a Canadian setting, associations between area income, immigrant density, and income inequality in relation to PTB and SGA birth. Previous studies have not considered the joint influence of these neighbourhood characteristics. We build on and extend previous research documenting interactions between area context and race [5,6,8,15,17,18,20,21,23]. Our results indicate that, independent of individual maternal characteristics, the influence of area context varies according to different social markers.

Our results are consistent with studies demonstrating an adverse influence of high area deprivation on PTB [1-6,8] and SGA birth [3-5]. Though area poverty was in our study more strongly associated with SGA birth than PTB, this finding was not observed in another study evaluating these same two birth outcomes [5]. The literature suggests PTB may be related to stress, possibly to a greater extent than SGA birth [44,45], while SGA birth may be more strongly related to lifestyle factors such as tobacco use [46,47]. Thus, the stronger associations with SGA birth may be related to the lower material/social resources present in deprived neighbourhoods, which may predispose less favourable behaviours among mothers. Our stratified analyses indicated that area poverty was more strongly associated with SGA birth among mothers with low compared to high education, this finding possibly being due to a greater level of unfavourable health-related behaviours in socio-economically vulnerable populations [48]. Our stratified analyses also indicated that area poverty was associated with PTB and SGA birth among Canadian-born but not foreign-born mothers. A separate study reported an association between area income and both PTB and full-term low birthweight (a proxy for SGA birth) among long-term residents but not recent immigrants of Toronto, Canada [7], in line with our findings. Foreign-born mothers, potentially healthier because of the selective process of immigration [7,49], may be more resilient than native-born populations to area deprivation. Other studies evaluating the influence of area disadvantage according to race (not maternal birth place) have not yielded consistent results [5,6].

We found a protective association between high immigrant density and PTB, and the magnitude of the association was greater than for area poverty. This finding is in line with research documenting fewer adverse mental health outcomes in areas with higher minority populations [50]. Stratified analyses, however, showed that high immigrant density was protective for PTB among Canadian-born mothers, but *unfavourably *associated with PTB among foreign-born mothers. This intriguing finding has not been previously reported. A study restricted to US Black mothers did, in comparison, find an elevated odds of PTB for racially concentrated neighbourhoods among mothers that were US-born but not those foreign-born [15]. The authors did not, however, account for neighbourhood income. Hence it is difficult to know the extent to which racial concentration may be a marker for poverty in the study. What can explain the interaction observed in our study? The stronger association with PTB than with SGA birth suggests that immigrant density may be operating through stress mechanisms, assuming PTB is more strongly linked to stress [5]. Thus, high immigrant density may be associated with conditions that reduce stress in native-born mothers, but increase stress in foreign-born mothers. Such conditions might arise if, for example, employment opportunities were greater for native- than for foreign-born individuals due to prejudice or network integration. The immigrant composition of Québec, with ties to French-speaking source countries, might also be driving these results although the underlying pathways are unclear.

We also found high immigrant density was associated with greater odds of PTB among mothers with high but not low education. Educated mothers might more readily access benefits conferred by high immigrant density (*e.g*., employment opportunities). Other studies, mainly focussing on segregation, have considered racial class but not education as a modifier of neighbourhood effects. Maternal education is generally treated as a control variable and not as a potential modifier [17,18,20]. Studies are, however, unclear on whether ethnic segregation is protective [16,19] or harmful [5,15-18] for PTB. An issue that complicates comparisons of studies is the wide spectrum of segregation measures that have been studied. However, when we compare our results to other studies that used a percentage-based indicator for segregation, results are still contradictory [5,15,17]. Thus, immigrant density in Québec may not be comparable to racial segregation in the United States.

While non-stratified models suggested that immigrant density may be protective for PTB, its associations with SGA birth were *unfavourable*. Our findings are in line with research supporting an adverse influence of ethnic segregation on SGA birth [5,16,22]. Unlike PTB, however, stratified analyses did not support a differential influence according to education.

Evidence for a contextual influence of income inequality was weaker than for area poverty and immigrant density, in line with research showing income inequality has a weaker influence in small areas compared to larger places [27]. Nevertheless, our results suggested an unexpectedly *protective *association, somewhat more for SGA birth than PTB. Protective associations between income inequality and adult outcomes have been observed by others [51-54], but not for birth outcomes. The sparse research performed thus far on birth outcomes supports an adverse association between high income inequality and PTB [23,28] or low birthweight [26]. Income inequality in Québec may be a characteristic of affluent neighbourhoods, affluence itself being associated with favourable birth outcomes [54]. Income inequality could also be a proxy for other unrelated neighbourhood factors favourably associated with birth outcomes. We used the CV, an indicator sensitive to the upper ends of the income spectrum [55], and associations could be different with indicators sensitive to other parts of the income spectrum. Two competing theories in the income inequality literature may also be considered [23,56]. These include the "neo-materialist" pathway which posits that underinvestment in material capital in less equal communities translates into lost opportunities and possible adoption of harmful health behaviours, and the "psychosocial" pathway which posits that relative comparisons experienced by less well-off individuals in unequal communities causes stress resulting in biologic effects [23,56]. These mechanisms suggest affluent neighbourhoods with high income inequality might have higher material capital (*i.e*., "neo-materialist" pathway) that would be expected to be beneficial for birth outcomes. Alternatively, neighbourhoods favouring environments in which striving for better life conditions is perceived positively (*i.e*., psychosocial pathway) may also be beneficial for birth outcomes.

Our results raise the possibility that the influence of income inequality on SGA birth might be more protective among mothers with high education. The protective influence of income inequality may more readily translate into health benefits when socio-economic status is high. The only comparable study evaluating the influence of income inequality according to class found an adverse influence on PTB among non-White mothers in the United States [23]. Other studies examining effect modification have focussed on adult outcomes [57-59].

This study is subject to limitations. We could not account for tobacco use and income which may confound or mediate relationships, although such factors do not fully account for area effects [1]. Statistical power was lower in stratified analyses for foreign-born mothers, due to smaller sample size. Our contextual indicators may be proxies for underlying factors that we cannot pinpoint with certitude. Although we examined several attributes of areas, we did not examine other dimensions such as segregation that could also contribute to adverse birth outcomes. Our indicator of income inequality was calculated from census data, which may not be as accurate as tax data [60]. We do not know length of residence in neighbourhoods, and the selection of mothers into neighbourhoods based on individual characteristics cannot be ruled out. The administrative boundaries that we used might not be the best level for the analysis of contextual effects [35]. Research indicates that the use of larger areas such as CLSCs might attenuate measures of contextual effects, however, these areas facilitate the study of income inequality [61]. Last, the extent to which results are generalizable to other non-US settings is unknown.

## Conclusion

This study demonstrates that the association between area factors and adverse birth outcomes in Québec is modified by maternal birthplace and education. Some studies have found race interacts in a similar manner, and our results indicate that social class indicators other than race influence this relationship. While both individual and area-level characteristics need to be accounted for in public health initiatives addressing adverse birth outcomes, there may be a further need to consider the interaction between these factors.

## Abbreviations

CLSC: local community service center; CV: coefficient of variation; GEE: generalized estimating equations; PTB: preterm birth; SGA: small-for-gestational age.

## Competing interests

The authors declare that they have no competing interests.

## Authors' contributions

NA designed the study. NA and JG reviewed the literature. NA guided and JG performed the data analyses. NA and JG interpreted the results and wrote the manuscript. MD provided structured neighbourhood-level data, helped interpret results and revised the manuscript for important intellectual content. All authors read and approved the final version of the manuscript.

## Pre-publication history

The pre-publication history for this paper can be accessed here:


